# Glycan Utilisation and Function in the Microbiome of Weaning Infants

**DOI:** 10.3390/microorganisms7070190

**Published:** 2019-07-04

**Authors:** Starin McKeen, Wayne Young, Karl Fraser, Nicole C. Roy, Warren C. McNabb

**Affiliations:** 1 Food Nutrition & Health, AgResearch, Grasslands Research Centre, Private Bag 11008, Palmerston north 4442, New Zealand; 2 Riddet Institute, Massey University, Private Bag 11222, Palmerston North 4442, New Zealand; 3 High-Value Nutrition National Science Challenge, Auckland 1023, New Zealand

**Keywords:** polysaccharides, complementary feeding, microbiome, glycobiology discipline

## Abstract

Glycans are present exogenously in the diet, expressed and secreted endogenously by host cells, and produced by microbes. All of these processes result in them being available to the gut microbiome, firmly placing glycans at the interface of diet–microbe–host interactions. The most dramatic shift in dietary sources of glycans occurs during the transition from the milk-based neonatal diet to the diverse omnivorous adult diet, and this has profound effects on the composition of the gut microbiome, gene expression by microbes and host cells, mucin composition, and immune development from innate towards adaptive responses. Understanding the glycan-mediated interactions occurring during this transitional window may inform dietary recommendations to support gut and immune development during a vulnerable age. This review aims to summarise the current state of knowledge on dietary glycan mediated changes that may occur in the infant gut microbiome and immune system during weaning.

## 1. Introduction

During weaning, infants’ primary source of dietary carbohydrates (glycans) transitions from mammalian milk-derived oligosaccharides and glycoproteins in breast-milk and/or animal-milk derived formula, to plant-derived polysaccharides in complementary foods. These glycans escape digestion in the small intestine of infants, becoming available as an energy source for the unstable and evolving gut microbiota, and this can influence gut and immune system development. Human and ruminant milk oligosaccharides are well characterised, and significant attention has been given to their interactions with the infant microbiome and immune system. However, less is known about the diversity of plant-derived glycans and their interactions in the infant gut. Evidence from dietary interventions with plant glycans in adults and in vitro studies provide a basis for predicting their prebiotic and immune-modulatory properties in weaning infants, but the acute responses of a microbiome that is adapted to mammalian glycans being exposed to plant glycans has not yet been investigated. This review aims to summarise how microbial community structure, gene expression by microbes and host cells, mucosa, and immune development in infants may be altered during dietary transition from milk to a combination of plant and animal glycans. 

## 2. Glycans: Sources, Structures, and Functions

### 2.1. Sources: Mammalian, Plant, Microbial

The human gut glycome encompasses exogenous glycans derived from diet, endogenous glycans expressed by host-cells and in secretions, microbial glycans, and viral glycans, all of which interact with each other. Glycans include conjugated glycoproteins and glycolipids, which are classified as either O-linked (attached to serine or threonine residues) or N-linked (attached to asparagine), attached to cell surfaces as the glycocalyx, or unconjugated oligoglycans, which are often found in plants and fungi [1]. Mammalian-derived glycans expressed in breast-milk, mucins, and on host cells, such as gut epithelial cells, are distinct from plant-derived glycans, which exist in tremendous unquantified diversity, and are subject to substantial modifications in structure and function during processing [2]. Microbes also express a diverse and unique array of glycans, which may be regulated in part by diet and environmental conditions in the gut. Some microbial glycans seem to be conserved throughout closely related genetic lineages, while others are present on extremely distant relatives, which may in part be due to lateral gene transfer between microbial species [2]. Enveloped viruses also possess a heavily glycosylated envelope membrane, with both N- and O-linked glycosylation patterns that are involved in infectivity, viral particle formation, and immune evasion [3]. While viral glycosylation may play key roles in lateral gene transfer, immunity, and the glycome of the infant gut, this will not be discussed in depth, due to the relatively little research in this area and the lack of clear linkages between dietary glycans and viral glycosylation. 

### 2.2. Structures and Functions

Biosynthesis of glycans occurs as a post-translational modification that takes place in the endoplasmic reticulum (O-linked) and Golgi apparatus (N-linked) of cells from all branches of life [2]. Glucose and galactose monomers make up most plant- and animal-derived glycan monosaccharides, however plant glycans also contain lower abundances of fructose, arabinose, rhamnose, galacturonic acids, and hexuronic monomers, creating diverse complex structures [4]. Various linkage types and branching structures add higher complexity in glycan structures, which dictates potential utilisation and metabolism by the host or microbiota. Terminal glycan linkages and modifications that are frequent subjects of investigation are sialylation and fucosylation, which have been implicated in the functional properties and recognition specificity of mammalian-derived glycans. Sialylation and fucosylation modifications are considerably less common in plant glycans.

Glycans are a source of carbon for microbes, and are crucial to recognition, signalling, and epigenetic regulation between host cells and microbes, which implicates them in a range of immunological and metabolic disorders [5]. Developing interventions and therapeutics that target glycan expression by hosts or microbes is complicated by their diversity and complexity, and hindered by a poor understanding of structure function specificity of both glycan motifs and enzyme cohorts employed by microbes [6,7]. Despite these limitations, interventions with dietary glycans, sometimes referred to as prebiotics, have been proposed for congenital disorders of glycosylation, which manifest as a range of immunological deficiencies and irregularities [8], as well as in treating metabolic and digestive disorders [9]. 

## 3. Dietary Glycans in the First 1000 Days

### 3.1. Human and Ruminant Milk Oligosaccharides

The milk-based diet of the first 4 to 6 months of human life contains mammalian-derived unconjugated glycans as the non-digestible oligosaccharide fraction of human milk (human milk oligosaccharides, or HMOs), and/or ruminant-derived oligosaccharides (ruminant milk oligosaccharides, RMOs) in infant formula. In breast-milk free unconjugated oligosaccharides are the third most abundant component, but glycoconjugates such as glycoproteins, glycopeptides, and glycolipids also play an important role in signalisation and defence against pathogens [10]. Concentrations of oligosaccharides in ruminant milk formulas vary by species, breed, lactation phase, and processing. Overall concentrations of bovine oligosaccharides have been found to be lower in infant formulas than in colostrum, mature milk, skim milk, or homogenised milk [11]. The microbiome of breast-fed infants is distinct from formula-fed infants, and while these differences diminish with age and the transition to an adult diet, the effects on the immune system may remain [12]. Supplementation of infant formulas with simple oligosaccharides (galactooligosaccharides, fructooligasccharides, and polydextrose) shifts the infant microbiota to more closely resemble that of the breast-fed infant, demonstrating increased abundance of *Bifidobacterium*, however different species and strains are promoted and long-term clinical outcomes have proven difficult to determine from recent birth cohorts [13,14,15,16].

**Figure 1:**
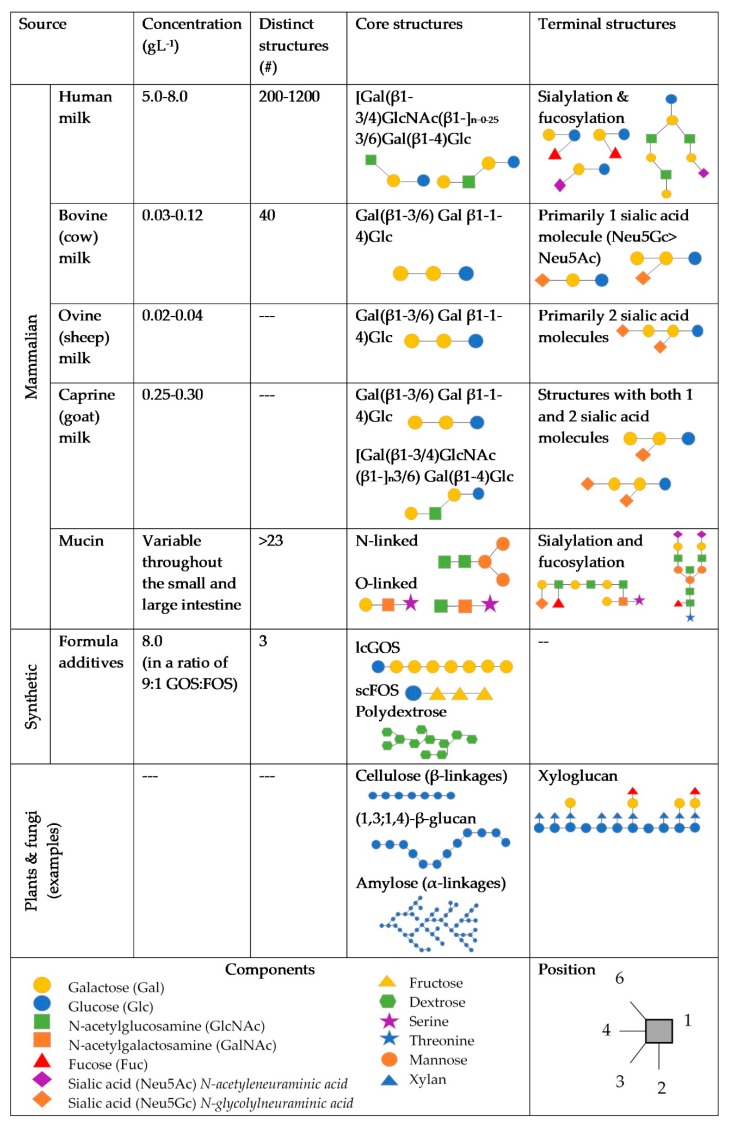
Dietary and endogenous glycans potentially found in the infant gut. References are included in the text.

It has been proposed that differences in concentration and diversity of glycans and between terminal structures on RMOs in infant formula may be primary factors affecting the microbiome composition and immune system. RMOs exist in lower concentrations compared to HMOs [17,18,19], with fewer unique structures having been identified [20,21], as shown in Figure 1. RMOs also have higher ratios of non-fucosylated neutral and sialylated structures, compared to HMOs [19,21,22,23,24]. Of these sialylated oligosaccharides, bovines express more Neu5Gc, which may be associated with inflammation [25], whereas humans express Neu5Ac [26]. Caprine milk has been found to contain the most similar profile and concentration of oligosaccharides to human milk, compared to other ruminant milks [27], however research in RMOs, particularly caprine and ovine, is sparse.

Recent studies have focused on the independent effects of fucosylated HMOs compared to sialylated HMOs on the infant microbiome. Natural differences in fucosylation of HMOs between and within lactating mothers, based on Secretor (Se) and Lewis blood group (Le) classification, allows the effects of fucosylation on the infant microbiome and immune system to be explored without interventions [28]. Protective benefits of fucosylated HMOs from Se mothers on the microbiomes of Caesarean born infants, which are otherwise depleted in Bifidobacteria and enriched in enterococci, has provided additional evidence to support efforts to fucosylate synthetic GOS for formula supplementation [29]. This reductionist approach may neglect the benefits that diversity and complexity provide to the infant gut. However, the roles that fucosylation and sialylation play in microbial recognition of substrates and host recognition of harmful substances are fundamental to glycobiological interactions in the infant gut.

### 3.2. Plant-Derived Glycans

As infants begin consuming solid foods, cereals and plant-based purees are typical first foods. Plant cell walls are complex composites of structurally diverse glycans and glycoconjugates that vary based on cell types, conditions during plant development, environmental exposures, processing, and culinary preparation. The most abundant and widespread elements of cell walls, cellulose, xyloglucan, heteroxylan, heteromannan, β-glucan, and pectin, are well characterised [4]. However, discrete structural details that affect purity, molecular weight, and solubility still need to be elucidated. These details affect binding capacity to enzymes and carbohydrate receptors, subsequent fermentability, and interactions with immune cells [4,30]. Several well characterised glycans have been investigated in in vitro digestions and fermentations, providing evidence that the rheology of food components effects microbial usage and consequent composition [31,32].

Complex dietary fibres and mixtures of various glycans also alters microbial utilisation patterns. In an in vivo study comparing infant cereals with varying ratios of complex to simple carbohydrates, increased complexity led to significantly higher butyric acid and secreted IgA, and lower faecal pH, despite no significant difference in faecal counts of *Bifidobacterium*, *Lactobacillus*, Enterobacteriaceae, *Enterococcus*, *Clostridium*, or *Bacteroidetes* [33]. When presented in a mixture, fibres are also utilised more slowly [34], which may partially explain the increased butyrate production as microbes switch from utilising monomers released from the fibre to the cross-feeding of intermediary metabolites, such as lactate and acetate, and so produce butyrate [35,36]. Increasing complexity of mixtures of non-digestible substrates also correlates with species diversity of microbiota in vitro, without significant alterations at the phyla and family level [37]. However, the effects of mixing plant-derived glycans with mammalian-derived glycans, is still to be characterised.

## 4. Microbiome

### 4.1. Neonatal Microbes and Dietary Glycans

The microbiome progresses from relatively low abundances of a few pioneer species in the neonatal gut, to a complex microbial ecosystem participating in competitive and symbiotic trophic networks, establishing ecological niches, and responding to unstable factors such as diet and immune responses. Colonisation of the infant gut may begin in utero [38], but species acquired during birth have a significant effect on microbiome composition, susceptibility to immune dysregulation, and interactions with dietary factors [39,40]. For instance, mode of delivery is a strong determinant of the effects of fucosylated milk glycans on the structure of the microbiome [29]. The microbiomes of caesarean-born infants who are breast-fed from non-secretor mothers with low-diversity non-fucosylated HMOs show higher relative abundances of aerobic environmental species, *Enterococcus* and *Akkermansia*, and decreased *Bifidobacterium*, compared to caesarean-born infants of Se+ mothers [29]. The microbiomes of vaginally delivered infants, which are enriched with anaerobic species from the maternal gut, were not associated with maternal Se status compared to caesarean born infants at 3 months of age. These findings show that fucosylation plays a protective role in the compromised gut of caesarean-born infants [29], which has been associated with increased risk for obesity and atopic disease [40,41]. Infants fed formula from ruminant milk, lacking fucosylated RMOs, display lower abundances of *Lactobacilli* and *Bifidobacterium* relative to increased abundances of *Clostridium*, *Bacteroides*, and members of the Enterobactereaceae family, which may result in a predisposition to colitis and atopic disease later in life [42]. In keeping with findings that the oligosaccharide profile of caprine milk is more similar than bovine milk is to human milk [27], the microbiomes of infants fed caprine milk formula may be more similar to that of breast-fed infants than those of infants who are fed bovine-milk derived formula. However, the only similarity observed was the predominance of *Ruminococcus gnavus* species within the *Lachnospiraceae* family in both caprine-milk and breast-fed infants, whereas microbiomes of bovine-milk-fed infants had a greater diversity of *Lachnospiracheae* [43].

### 4.2. Effect of Dietary Glycans at Weaning

The concentration of HMOs in breast-milk decreases over the course of lactation [44], and the fucosylation of HMOs by lactating mothers decreases around the time of introduction of solid foods to infants [28,45]. The effects of compositional differences during progressing phases of lactation on the infant microbiome are difficult to separate from increasing dietary diversity, requiring the application of in vitro investigations, in vivo population cohorts, or randomised controlled in vivo interventions to study. An in vitro investigation into the microbiome response to different cereal products showed a high level of inter-individual variation between faecal inoculum (complicated by age, feeding mode, and dietary diversity) [32]. The introduction of digested oats was correlated with an increase in *Veillonellaceae*, and relative abundance of *Bifidobacteriaceae* was significantly higher in samples provided digested rice compared to digested oats, sorghum, or wheat, indicating substrate preferences within families of glycan degrading microbes [32]. Comparing cohorts of infants raised in environments with different dietary habits provides further insights into microbiome variations. Children living in Burkina Faso consuming a diet rich in complex carbohydrates from cereal and legume sources had microbiomes enriched in Bacteroides compared to Italian infants (57% *vs*. 22%), and contained several specialised starch degrading species such as *Prevotella* and *Succinovibrio*, not found in the Italian infant cohort [46]. The Italian infants had microbiomes enriched in Firmicutes (63% *vs*. 27%), which were associated with diets high in protein and fats [46]. These differences are likely confounded by vertical transmission of microbiota from mothers, as well as environmental exposures, but the glycan degrading capacity of Burkinabe infant microbiota shows a strong correlation with high dietary glycan consumption. Despite variations in enrichment patterns at the species level, the universal trends in microbiome composition during weaning are increased abundances of *Bacteroides* and *Firmicutes (Clostridia)*, accompanied by a decrease of *Actinobacteria* (*Bifidobacterium)* and *Enterococcus* populations over time [47,48,49]. Importantly, it has been suggested that the most drastic changes in the microbiome structure and stability occurs with the cessation of breastfeeding [47], though this effect has not been explored in formula-fed infants.

### 4.3. Species Characteristics

Shifts in relative abundances of glycan degrading microbial species and expression of glycan utilising enzyme systems during dietary transitions highlights roles and characteristics of relevant species within the greater microbial community. Many microbial species cannot utilise glycans longer than one or two sugars, but rather depend on extracellular release of monomer subunits from “sharing” species of glycan degraders for energy, or fermentative by-products of glycan metabolism from “selfish” microbes [50]. For instance, many members of the *Firmicutes* and *Actinobacteria* phyla adhere tightly to insoluble plant wall fibres, doing some of the initial processing that then releases shorter glycan chains for utilisation by other microbes [51]. The potential to degrade plant-derived glycans has been found in the neonatal microbiota using shotgun metagenomics. However, expression of these enzymes likely does not occur until plant-derived glycans are consumed and reach the microbiota, as has been demonstrated for resistant starch [52]. Many mucin degrading microbes have the metabolic flexibility to switch between degrading exogenous dietary glycans and endogenous glycans from mucins, increasing survival prospects during times of low substrate availability [53]. 

### 4.4. Trophic Networks, Hierarchies, and Biogeography 

The ecosystem of the gut microbiome changes longitudinally throughout the gut, radially from the epithelium to the lumen, and temporally. Gas gradients, pH, and nutrient distribution all interact to shape the biogeography and dynamics of the microbial ecosystem. Primary degraders of glycans are found in higher abundances in the proximal colon, whereas secondary degraders and proteolytic bacteria increase distally, along with pH, which may be in part due to the distal availability of proteins released from hydrolysis of glycoproteins earlier in the colon [54]. At cross-sections of the colon, an oxygen gradient originating from tissue oxygenation descends from the gut epithelium, through the mucosa, into the centre of the lumen [55]. In healthy adults, average partial pressure of oxygen values in the lumen are extremely low (~1 mm/Hg) [56], conducive to anaerobic species, which tend to be saccharolytic and produce short chain fatty acids through fermentative metabolism in the anaerobic environment [57]. Microbiota that adhere to the gut mucosa show decreased abundance of genes implicated in carbohydrate metabolism, based on inferential PiCRUST analysis [55], however *Bacteroidetes* tend to be under-represented in faecal samples compared to colonic mucosal biopsies [58]. In the infant gut, oxygen levels throughout the colon are higher, corresponding with colonisation by aerobic and facultative anaerobic species, which are predominantly proteolytic [59,60]. Oxygen consumption by early oxygen tolerant species, of the *Proteobacteria* and *Actinobacteria* phyla, may help facilitate the colonisation of saccharolytic obligate anaerobic species [61]. Interpretation of microbiome data from faecal samples, either directly analysed or used in in vitro fermentation experiments, requires caution because such samples do not accurately reflect the whole microbial community [62].

## 5. Dietary Glycans Influence the Gut Mucosa

The gut mucosa forms a dynamic, bi-layered, physical and immunological barrier between the gut epithelium and microbiota residing in the lumen, allowing for communication via metabolites, selective translocation of microbes through the gel-like matrix, and providing a stable source of energy for glycan degrading microbes and protection for the gut epithelium. 

### 5.1. Mucin Production and Glycosylation

Goblet cells in the small and large intestines produce high-molecular weight glycoproteins called mucins, the expression and structure of which can be altered epigenetically in response to dietary factors and stressors such as infection. Over 20 different mucin genes (*MUC1-21*) have been identified, demonstrating tissue-specific expression; *MUC2*, *MUC3A/B*, *MUC12*, *MUC13*, *MUC15*, *MUC17*, and *MUC20* are expressed in gut tissues [63]. Mucins that adhere to epithelial cells are predominantly N-linked, whereas gel-forming crosslinked mucins are predominantly O-linked. These distinct terminal glycosylation patterns influence susceptibility to degradation and turnover [63]. O-linked glycosylation plays a role in determining whether disease will be promoted or averted [64], in part due to the protection that glycosylation provides from degradation by the host, while supporting numerous commensal microbial species [65].

### 5.2. Composition of the Mucosa

The mucosa interacts with the digesta moving through the lumen, meaning that non-digestible carbohydrates alter the pH, viscosity, and glycan composition of the mucosa. Insoluble fibres can be a mechanical irritant that erodes the mucosa, stimulating the release of mucins into the lumen [66]. In a gnotobiotic murine model, goblet cells were found to release more mucins with a higher negative net charge in a murine model that had adapted to a high fibre diet, which may contribute to a higher viscosity mucus [67]. The increase in mucin production may be modulated by butyrate production during the fermentation of fibres, which has a limited dose-dependent effect on mucus production in murine models [68]. The solubility and rheology of the source of dietary glycans are likely to govern the effects in the mucosa. Maintaining elasticity, changeable rheology, and self-repair mechanisms is crucial for the translocation of small molecules such as metabolites, and immobilising pathogens [69]. However regular turnover of the mucosa, via sloughing, foraging, and peptide degradation, is also associated with gut health [63], highlighting the delicate homeostatic balance that both dietary and endogenous glycans are involved in.

## 6. Glycan Utilisation Systems by Infant Microbiota

The collective microbiome expresses a tremendous diversity of glycan degrading enzymes compared to mammals. Glycan utilising enzymes expressed by saccharolytic bacteria are called Carbohydrate-Active Enzymes (CAZymes), which include glycoside hydrolases (GHs) and polysaccharide lyases. The protein fold of the enzyme, target linkage, catalytic apparatus, and mechanism of glycan degradation are conserved within families, but substrate specificity can be highly variable within species and subspecies [70]. Determining enzyme structure and positioning relative to the cell wall can be partially indicative of substrate specificity, selfish or sharing mechanisms, mutualistic behaviour, and roles within trophic networks [71]. Metabolic flexibility and the ability to apply glycan degrading mechanisms to both host-derived glycans from mucins, as well as exogenous dietary glycans, is a common feature that contributes to the survival and persistence of species during dietary transitions and stress. Complementary glycan degrading enzymes are also a feature of some of the key mutualistic relationships among commensal microbes that shift in populations during the transition from mammalian glycans to diverse exogenous sources.

The milk-oriented microbiome of breast-fed infants is dominated by *Bifidobacterium*, likely due in part to the ability of *B. longum* subs. *infantis* and *B. bifidum* to utilise HMOs as their sole carbon source, using a variety of enzymatic mechanisms [72]. A taxonomic marker of the Gram-positive anaerobic generalist *Bifidobacteriaceae* family is the “bifid shunt,” a highly efficient saccharolytic metabolic pathway [73]. However, species and subspecies express highly variable enzymes needed to channel specific diet- and host-derived glycans into the bifid shunt. As a genus, *Bifidobacterium* tends to share carbohydrate resources derived from both conjugated and unconjugated glycans, which may be due in part to expression of intracellular enzymes, extracellular enzymes, and secreted enzymes [74,75], many of which encode 2 α-sialidases, 5 α-fucosidases, 5 β-galactosidases, and 3 β-*N*-acetylglucosaminidases, which are activated during growth on HMOs. However, *B infantis* (which expresses an assortment of internalisation transporters for neutral and acidified HMOs [72]) and *B. breve* import intact HMOs and degrade them intracellularly. In contrast, *B. bifidum* species are more specific and divergent in their preferences and ability to utilise acidified glycans and degrade HMOs extracellularly [72,76]. The ability of *B. infantis* to utilise all structures of HMOs offers this species a significant ecological advantage over competing species, and its internalisation of monomeric subunits limits support for mutualistic species. Collectively, these aspects may offer insights as to why microbiomes of the breast-fed infant have been found to have lower α-diversity than formula-fed infants, despite consuming a greater diversity of glycan structures in human milk.

Additional enzymatic capacity that contributes to the survival of *Bifidobacterium* during dietary transitions is their ability to degrade mucins, which has been linked to sulfoglucosidase activity [77,78] and glycoside hydrolases [79], which are induced by mucin binding [64,80]. The ability of various strains of Bifidobacterium to utilise both N- and O-linked glycans has been attributed to the differential expression of a variety of extracellular and membrane bound GHs [81,82].

HMO utilisation by other gut bacteria is variable and not a widespread phenotype [71,83], though *Bacteroides fragilis* and *B. vulgatus*, prominent Gram-negative members of the *Bacteroidetes* phylum in the infant gut, are able to consume HMOs by engaging enzymatic machinery from mucin degrading pathways towards the degradation of milk-derived glycans [84]. Whereas *Bifidobacterium* prefers mammalian derived glycan to mucins and rely on cross-feeding interactions with *Bacteroides* to utilise plant-derived glycans [85], *Bacteroides* may prefer plant-derived glycans to mammalian-derived and host glycans. These preferences and mutualistic behaviours are demonstrated by co-culture experiments with specific strains of *Bifidobacterium* and *Bacteroides* on HMO or xylan substrate [84,85]. This type of mutualism and prioritisation of substrates may sustain *Bacteroides* populations until they are required for the utilisation of plant-derived glycans at the introduction of solid foods, while supporting Bifidobacterium populations as HMOs become scarce. Similarly, *Lactobacillus*, of the Gram-positive *Firmicutes* phyla, prefer the monosaccharide components of HMOs that are released by extracellular and secreted enzymes from *Bifidobacterium* [79,86]. *Bacteroidetes* and *Firmicutes* express polysaccharide lyases, including the well characterised transmembrane starch utilisation complex (Sus), which are orchestrated by polysaccharide utilisation loci (PUL) and Gram-positive PUL (gpPUL), respectively [87].

*Bacteroidetes* is the quintessential generalist saccharolytic phylum containing prolific glycan degraders such as *B. thetaiotamicron* and *Prevotella*. In *B. thetaiotamicron*, Sus has become the archetypal PUL, characterised by components involved in recognition, initial hydrolysis at the outer membrane, glycan translocation into the periplasm, further hydrolysis into monosaccharides, and transcriptional regulation [88]. Multiple pleiotropic regulators respond to different signals that collectively govern how *B. thetaiotamicron* prioritises various polysaccharides for use. For instance, the detection of monomeric fructose regulates catabolism of both monomeric fructose and polymeric fructans [89], whereas a transcriptional activator responds to arabinan and a transcriptional repressor responds to arabinose [90]. This concept of pleiotropic detection and signalling may be extrapolated for investigations into similar and competing species involved in the hierarchical utilisation of glycans. Polymer length and branching complexity of plant-derived glycans are contributing attributes that determine microbial preferences and utilisation within these trophic networks, supporting the theory that diverse dietary glycan structures support diverse Bacteroides species [91].

The complexity of feedback loops between detection, signalling, transcription, and expression of enzymes, polysaccharides, and metabolites in response to substrate availability is further complicated by host interactions that are mediated by glycosylation patterns. α-Fucosylation on the host gut epithelium during development is associated with the recognition of l-fucose by *B. thetaiotamicron*, which indicates that microbial foraging of fucose from host glycans triggers increased production by the host, via *FUT2* (a1,2-fucosyltranferase enzyme). Interestingly, there is also an increase in *FUT2* transcriptional activity during bacterial infection, which may support mutualist populations during times of stress [92].

Members of the *Firmicutes* phylum are the predominant butyrate producing bacteria in the human gut, and include *Faecalibacterium prausnitzii, Roseburia* spp., *Eubacterium rectale*, *Eubacterium halii*, and *Anaerostipes*. However, relative to *Bacteroidetes*, little is known about their carbohydrate utilisation pathways. Pan-genome analysis of *Roseburia* spp. and *Eubacterium rectale* revealed a high degree of substrate specificity by species, linked primarily to ABC transport systems and regulatory genes [87,93]. *Firmicutes* are not primary glycan degraders but are efficient at capturing glycan monomers [94]. In keeping with the substrate specificity at the strain level, only certain species of *Firmicutes* have been found to utilise mucin as a sole carbon source, such as *Ruminoccus gnavus* ATCC 29149, whereas other species of *Ruminococcus* can only assimilate released monomers [95]. Relative abundance of *Firmicutes* increases over the course of weaning (between 4–12 months of age) [47].

## 7. Microbial Biosynthesis of Glycans

Microbes are underexplored repositories of glycans but are known to build structural polysaccharides, polysaccharide capsules, lipopolysaccharides, and exopolysaccharides collectively known as microbially produced glycans (MPGs). These MPGs have multiple symbiotic roles in the gut that contribute to overall fitness [92,96]. They have been implicated in phage evasion [97,98], immunomodulation of host, inhibition of phagocytosis (complement killing), and inhibition of antibody generation/deposition [99]. In addition, they function as bacterial nutrients [100], and contribute to the exclusion of pathogens [101]. It is unknown how MPGs influence the metabolism of dietary glycans: they may help activate metabolic pathways and enzyme expression for exogenous glycan degradation, but they may also be preferred as substrate, as has been found with fructan exopolysaccharide produced by *Lactobacillus* [102].

Microbial glycosylation and secretion of glycans is best understood for pathogenic species, which also utilise glycans for recognition, signalling, and survival strategies, such as mimicking host glycans to evade host immune recognition [1]. Commensal expression and secretion of glycans is poorly understood in gut associated microbes. The presence of monomer subunits that correspond with genetically coded biosynthetic mechanisms allows for monomers from catabolism to be recycled into extracellular glycans, with further roles in complex feedback loops that link diet with microbes and hosts. Four broad biosynthesis pathways are known to exist for MPGs. A well characterised pathway is the Wzy-dependant mechanisms in *E. coli* where glycosyltransferases in the cytoplasm assemble repeat oligosaccharides, which are then polymerised into longer chains on the cell surface (Gram-positive bacteria), or in the periplasm before export to the cell surface (Gram-negative bacteria) [98]. In ABC transporter-dependant synthesis the entire glycan chain is polymerised in the cytoplasm [103]. Synthase-dependant and glucansucrase/fructansucrase-dependant pathways produce less complex glycans, with only one or two monosaccharides [104]. 

The coordination of glycan-derived substrate availability with biosynthesis of MPGs is best understood for *B. thetaiotamicron*. *Bacteroides* rely on the ability to synthesise phase variable surface glycans for immune evasion and successful colonisation. This is demonstrated by *B. fragilis* mutants which have limited ability to compete to colonise gnotobiotic mouse gut when defective in four or seven of eight capsular glycans, and are unable to colonise as an acapsular mutant [105]. Transcriptional regulation of PUL is associated with multiple trans-envelope signalling switches, regulated by the availability of monomer subunits, which in turn regulate the biosynthesis by extracellular capsules in a coordinated manner [106]. This coordinated mechanism implicates glycans as a link between diet, microbial physiology, and host response, particularly in the developing gut.

## 8. Dietary Glycans in Immunity

**Figure 2:**
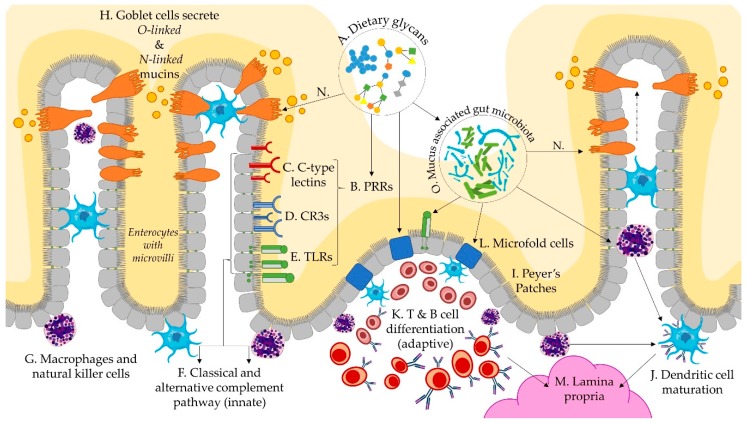
Schematic representation of dietary glycan and immunological interactions via the infant gut microbiome. Dietary glycans (**A**) interact with pattern recognition receptors (**B**), including C-type lectins (**C**), pleiotropic complement receptor type 3 (**D**), and toll-like receptors (**E**), which activate immunological pathways (**F**) via macrophages (**G**), natural killer cells, and dendritic cells. Goblet cells secrete mucins (**H**) into the lumen and sample lumenal material (**N**). Peyers patches (**I**) are mucosal immune structures that facilitate dendritic cell maturation (**J**) and differentiation of B and T cells (**K**), by way of lumen facing microfold cells (**L**), which contribute to the differentiation and transfer of immune cells to the sub-epithelial lamina propria (**M**). Sampling of lumenal material by goblet cells (**N**) is an additional mechanism by which mucosa associated microbes (**O**) facilitate the development of immune tolerance.

Glycans are the at the core of immunological interactions between host cells, microbes, and the mucosal matrix, as depicted through interactions in Figure 2A–O. Multiple pathways of immunomodulatory action have been identified, leading to the reclassification of functional polysaccharides as secondary metabolites [107,108] and biological response modifiers (BRM) [109]. During early life, the infant immune system transitions from a reliance on innate immunity to adaptive immune responses, characterised by a tolerance for commensal organisms, and targeted immune responses to pathogens. Diversifying dietary glycans (Figure 2A) with direct and microbial BRM activity during weaning may support this transition by supporting gut maturation, facilitating tolerogenic immunity, and linking innate and adaptive immune responses. Glycans are known to play a multitude of other critical roles in immunity and immune system development than discussed here [110], but have thus far not been associated with alterations in diet.

Structure, origin, purity, molecular weight, and solubility of dietary glycans (Figure 2A) affects the binding capacity to pattern recognition receptors (PRRs) (Figure 2B) on epithelial cells, macrophages, and dendritic cells with direct immunomodulatory functions [30]. Gut epithelial cells lining the mucosa express both glycans and glycan-binding receptors in the form of PRRs, such as C-type lectins (Figure 2C), pleiotropic complement receptor type 3 (CR3) (Figure 2D), Toll-Like Receptors (Figure 2E), and scavenger receptors [5,111]. These types of receptors detect exogenous dietary glycans, activating both the classical and alternative complement pathways (Figure 2F) [109]. The signalling cascades that they initiate are variable and pleiotropic according to binding and adhesion capacity and intracellular signalling cascades [112]. Mitogen activated protein kinases are found in macrophages, B-lymphocytes, and natural killer cells (Figure 2G) which are active in both innate and acquired immunity, indicating a potential role of dietary glycans in modulating both innate and adaptive immune responses.

The specificity of immune factors towards pathogens and tolerance towards commensals is in part regulated by specialised epithelial cells and goblet cells (Figure 2H). Epithelial cells are able to secrete soluble mediators which interact with lymphoid structures, such as Peyers patches (Figure 2I), where adaptive immune responses are initiated on antigen presenting dendritic cells (Figure 2J), macrophages, or effector T-cells (Figure 2K) [113,114]. Microfold cells (Figure 2L) are fucosylated, mediated by fucosyl transferase genes (*FUT1*), within the gut-associated lymphoid tissue of Peyers patches and in the mucosa-associated lymphoid tissue of the gut, which contribute to the transport of microbes and particles from the lumen to the lamina propria (Figure 2M), and subsequent initiation of mucosal immune responses [5,115,116]. Goblet cells are also able to sample luminal material during mucus secretion (Figure 2N) and transfer the antigens to lamina propria dendritic cells [117]. Collectively, these interactions shape the gut mucosa towards a more tolerogenic mucosal phenotype, which is associated with decreased risk for atopic and inflammatory bowel disease [114]. 

Prebiotics and probiotics (Figure 2O) have been associated with improved dendritic cell differentiation and maturation. However, when lipopolysaccharides were removed from HMO substrate, HMOs were found to have no effect on dendritic cell differentiation and maturation in vitro [118]. Lipopolysaccharides expressed by microbes play a central role in the induction and regulation of immune responses and are increasingly implicated in linking and balancing adaptive and innate immune responses [119,120,121]. Multiple *Bacteroides* species, including *B. fragilis* and *B. thetaiotamicron*, express polysaccharides that induce regulatory T cells (T_reg_) [119,121]. *B. fragilis* expresses polysaccharide A which activates CD4+ T cells, resulting in a Th1 response, and correcting the Th2 cell skew seen in gnotobiotic mice [119]. *B. thetaiotamicron* generates numerous bacterial antigens with outer membrane vesicles that express sulfatases allowing them to access host immune cell, which promote inflammatory immune stimulation [120]. Recently, dietary factors have been found to regulate the expression outer membrane vesicle antigens: a high glucose diet reduced protein expression of a peptide that is both recognised by T cells and stimulates the differentiation into T_reg_ cells and effector T cells, with depletion of T_reg_ cells resulting in colitis [122]. The increased availability of glucose in the lumen from extracellular degradation of complex glycans may generate steady concentrations of glucose that regulate differentiation pathways of immune factors. While this is but one diet-mediated mechanism that has been elucidated, the concept of diet–microbe–immune mediated pathogenesis or disease aversion is pertinent to the developing immune system during dietary transitions. 

## 9. Summary

Expansion of glycan diversity, transitions in and mixtures of dominant glycan structures, and eventual elimination of formative dietary glycans creates a tumultuous environment with profound effects on the assembly and function of the weaning infant microbiome. Emerging evidence suggests dietary glycans drive the microbiome structure and modulate the expression of microbial and host glycans. Interactions between microbe/diet-glycans and microbe/host-glycans regulate gut epithelial development, immune response to pathogens, and transitions to adaptive tolerogenic immunity. Thus, intentional introduction of dietary glycans through complementary foods may present an opportunity to promote immediate and long-term immunity by influencing a network of interconnected carbohydrate-based transactions in the gut. However, the challenges of studying complex and nuanced glycomics are substantial, and further intensified by the rapid development of infants. Research in this field requires a combination of mechanistic insights gained through in vitro experimentation, and systems approaches to in vivo interventions, with specific consideration for the qualitative and temporal limitations of the samples that can reasonably be analysed from infants.
